# A transcribed ultraconserved noncoding RNA, Uc.173, is a key molecule for the inhibition of lead-induced neuronal apoptosis

**DOI:** 10.18632/oncotarget.6590

**Published:** 2015-12-13

**Authors:** Aruo Nan, Xinke Zhou, Lijian Chen, Meiling Liu, Nan Zhang, Li Zhang, Yuanwei Luo, Zhenzhong Liu, Lijun Dai, Yiguo Jiang

**Affiliations:** ^1^ State Key Laboratory of Respiratory Disease, Institute for Chemical Carcinogenesis, Guangzhou Medical University, Guangzhou, PR China; ^2^ Institute for Chemical Carcinogenesis, The Fifth Affiliated Hospital, Guangzhou Medical University, Guangzhou, PR China; ^3^ School of Public Health, Guangdong Pharmaceutical University, Guangzhou, PR China; ^4^ Laboratory Animal Center, Guangzhou Medical University, Guangzhou, PR China

**Keywords:** Uc.173, T-UCR, lead, neuronal apoptosis, Pathology Section

## Abstract

As a common toxic metal, lead has significant neurotoxicity to brain development. Long non-coding RNAs (lncRNAs) function in multiple biological processes. However, whether lncRNAs are involved in lead-induced neurotoxicity remains unclear. Uc.173 is a lncRNA from a transcribed ultra-conservative region (T-UCR) of human, mouse and rat genomes. We established a lead-induced nerve injury mouse model. It showed the levels of Uc.173 decreased significantly in hippocampus tissue and serum of the model. We further tested the expression of Uc.173 in serum of lead-exposed children, which also showed a tendency to decrease. To explore the effects of Uc.173 on lead-induced nerve injury, we overexpressed Uc.173 in an N2a mouse nerve cell line and found Uc.173 had an inhibitory effect on lead-induced apoptosis of N2a. To investigate the molecular mechanisms of Uc.173 in apoptosis associated with lead-induced nerve injury, we predicted the target microRNAs of Uc.173 by using miRanda, TargetScan and RegRNA. After performing quantitative real-time PCR and bioinformatics analysis, we showed Uc.173 might inter-regulate with miR-291a-3p in lead-induced apoptosis and regulate apoptosis-associated genes. Our study suggests Uc.173 significantly inhibits the apoptosis of nerve cells, which may be mediated by inter-regulation with miRNAs in lead-induced nerve injury.

## INTRODUCTION

Lead, one of the main heavy metals, is widely distributed in the environment. Lead exposure in the living environment harms human health; in particular, prevalence of lead poisoning is much higher in children than in adults. It has been suggested that lead can act as a kind of poison with strong neural developmental toxicity [1]. Even low-level exposure to lead can harm the developing central nervous system, and influence the ability of learning, memory and neural behaviors of children. Because of the incomplete development of the blood brain barrier in children, when blood lead accumulates to a certain level, it will induce irreversible injuries in the nervous system [2, 3]. The neurotoxicity of lead is associated with neuronal apoptosis. The excessive apoptosis of nerve cells may have a serious impact on normal development and differentiation of the central nervous system. Studies show that lead exposure can lead to apoptosis of nerve cells in human and experimental animals. In recent years, studies have indicated that long non-coding RNAs (lncRNAs) have a very important role in the process of carcinogenesis and damage induced by xenobiotics [4], but the mechanism of lncRNAs in lead-induced nerve injury has not yet been reported.

LncRNAs are a form of RNA whose length is more than 200 nucleotides. They themselves do not encode proteins but participate in the regulation of gene expression and various biological processes [5]. LncRNAs can activate or inhibit the expression of target genes by directly binding to the target genes, or be involved in histone modification or recruitment of transcription factors. LncRNAs also interact with microRNA (miRNA) as a kind of competitive endogenous RNA (ceRNA) to regulate the expression of target genes [6]. LncRNAs also inter-regulate with miRNA as ceRNA [7].

There are 481 ultra-conservative regions (UCRs), which share 100% identity among human, mouse and rat genomes [8]. The lncRNAs transcribed from these UCRs are known as transcribed ultra-conservative regions (T-UCRs) [9]. The functions of most T-UCRs are still unknown, but their high conservation and wide expression in various tissues suggest they have important biological functions. The changes of T-UCR expression irrespective of type or amount have proven closely related to the survival rate of human cancer patients, including chronic lymphocytic leukemia, colon cancer, primary liver cancer and neuroblastoma [10]. Moreover, studies show that some T-UCRs, especially Uc.73 and Uc.338, can promote the growth of cancer cells by acting as an oncoprotein [11]. The studies regarding T-UCRs mainly focus on their role in cancer, but some studies report that they could be potential therapeutic targets [12]. However, their functions in nerve injury induced by exogenous chemicals have not been reported.

In our previous study, we treated PC12 cells (a cell line derived from a pheochromocytoma of rat adrenal medulla) with lead acetate and made lncRNA chip and RT-qPCR (Reverse Transcript-quantitative PCR) analysis. We found Uc.173 was downregulated significantly among the differentially-expressed genes. To investigate the role of Uc.173 in lead-induced nerve injury, we tested the changes of the expression of Uc.173 in brain tissue, serum of lead-exposed mouse as well as in the serum of lead-exposed humans. We used N2a mouse nerve cells to further explore the effects of Uc.173 on neuronal apoptosis induced by lead. Our results suggest Uc.173 plays an important role in apoptotic inhibition in lead-induced nerve injury.

## RESULTS

### Establishment of a mouse model with lead-induced brain injury

**Figure 1:**
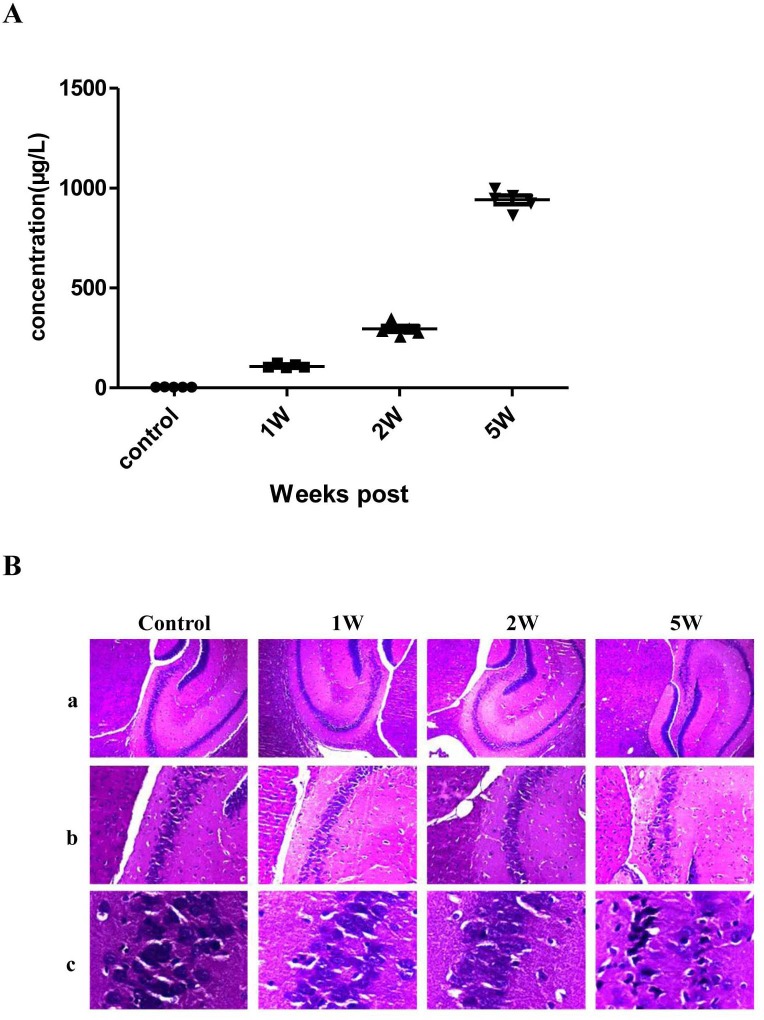
Blood lead concentration and hematoxylin-eosin (HE) staining of hippocampus tissue from a lead-exposed mouse **A**. shows the blood lead concentrations of the lead-exposed mouse at different time points. **B**. shows the HE staining of the hippocampus tissue of the lead-exposed mouse. In **B**., a, b and c represent the images amplified by 40-, 100- and 400- fold. As shown in the figure, obvious pathological changes are not observed in the control group and the 1 Week and 2 Week lead-exposure groups. But in the 5 Week lead-exposure group, significant pathological changes are observed which mainly exhibit as a granular layer of cells in the C3 region of the hippocampus which become sparse and the cell number is significantly decreased. A large amount of granular layer cells become thinner and longer and nuclei show obvious karyopyknosis.

To test the changes of expression of Uc.173 during brain injury induced by lead, we established a C57bl/6 mouse model of lead-induced brain injury. We used inductively coupled plasma mass spectrometry (ICP-MS) to monitor the blood lead concentration in mice. Compared with controls, the blood lead concentration increased slightly in Week 1 and Week 2 of the lead treatment while it increased significantly in Week 5 (Figure 1A). The reason for no significant increase in the first two weeks is that it takes time for lead to accumulate in mice. In Weeks 1, 2 and 5, the histopathological hematoxylin-eosin staining analysis of hippocampus tissues suggested that in early stages of lead exposure, brain tissues had no significant injuries, but showed remarkable pathological changes in Week 5 (Figure 1B) compared with controls. The elevation of blood lead level correlated with the degree of brain damage.

### Expression of Uc.173 in the serum of the animal model and lead-exposed population

**Figure 2:**
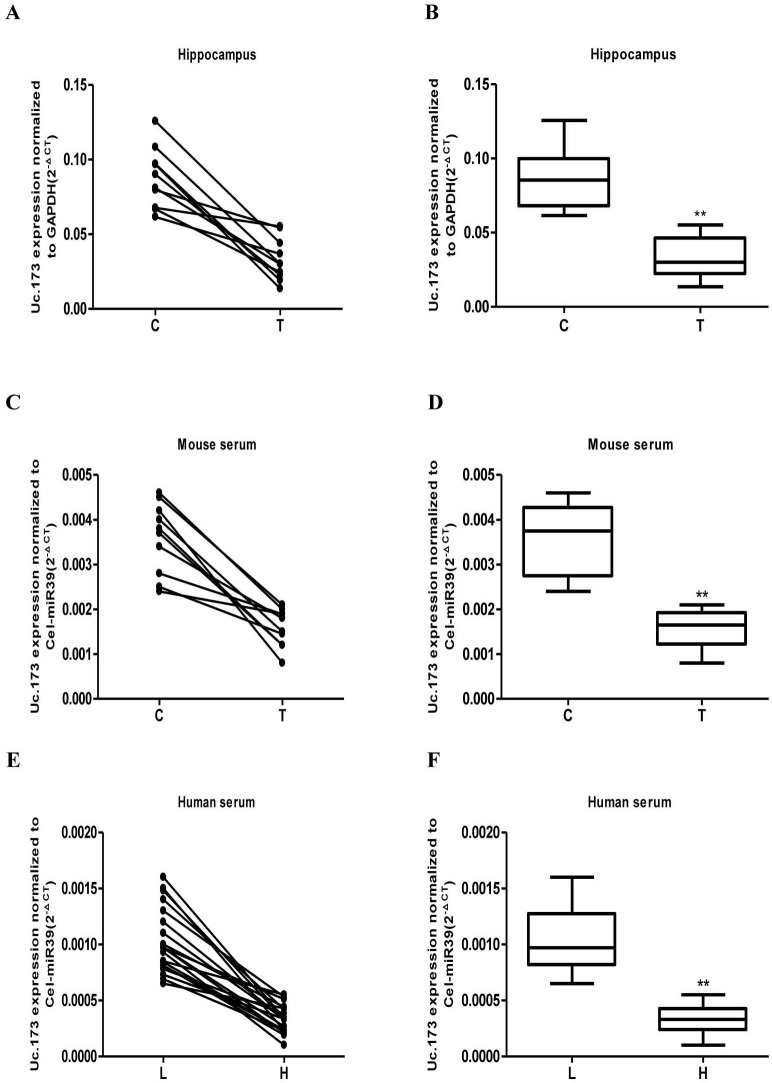
Detection of the expression of Uc.173 in hippocampus tissue and serum of lead-exposed mouse as well as the serum of lead-exposed population by qRT-PCR After being exposed to lead for five weeks, the expression of Uc.173 in hippocampus tissue (**A**. and **B**.), in mouse serum (**C**. and **D**.), as well as in lead-exposed human serum (**E**. and **F**.). C represents the control, T represents lead treatment (exposure), L represents low-lead population and H represents high-lead population. The internal control used in hippocampus tissue test was GAPDH. Relative quantification which applied Cel-miR 39 as an external control was used to evaluate expressional level of Uc.173 in the serum of mice and humans. 2^−ΔCT^ was used for relative quantification. ΔCT is the value of the CT value of Uc.173 subtracting the CT value of the internal (or external) control. B, D and F box diagrams represent the average level of Uc.173. A paired *t*-test was used in the animal model and a Mann-Whitney test was used in the human samples. ** represents highly statistically significant difference (*P* < 0.01).

We used RT-qPCR to test the expression of Uc.173 in the hippocampus tissues and serum of lead-exposed mice and in the serum of lead-exposed populations. Each group included ten mice. The animal model was successfully established after five weeks treatment of lead. Then we tested the expression of Uc.173 in mouse hippocampus tissues (Figure 2A and 2B) and serum (Figure 2C and 2D) and found the expression of Uc.173 was downregulated significantly (decreased by 2.6-fold) in lead-treated groups compared with controls. We also tested the level of Uc.173 in human serum. Since we mainly investigated the lead-induced injury in the developmental stage, we chose school-age children as study subjects. We firstly tested the blood lead concentration in children who lived in a selected district and were more likely to be exposed to lead, then we chose 25 children with low-level lead exposure as the low-lead group (less than 20 ug/L of blood concentration) and 25 children with a high-level lead exposure as the high-lead group (more than 80 ug/L of blood concentration). We collected serum samples to test the expression of Uc.173. The expression of Uc.173 was significantly downregulated (decreased by 3.1 folds) in the high-lead group compared with the low-lead group (Figure 2E and 2F).

### Expression of Uc.173 in lead-treated nerve cells

**Figure 3:**
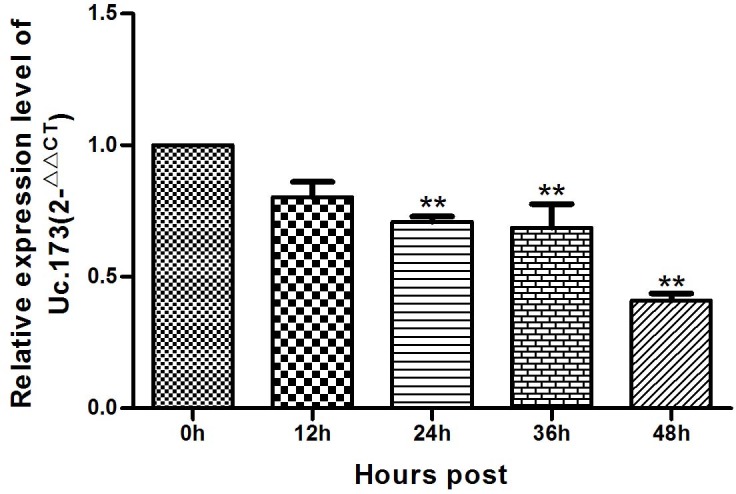
Expression of Uc.173 in N2a nerve cells of lead-exposed mouse 0 h, 12 h, 24 h, 36 h and 48 h represent the duration of lead exposure. Relative quantification (2^−ΔΔCT^ method) applying GAPDH as an internal control. Statistical analysis with applied one-way ANOVA. ** represents a highly statistically significant difference (*P* < 0.01). As shown in the figure, the expression of Uc.173 was downregulated after 24 h, 36 h and 48 h lead exposure (*P* < 0.01).

To study the effects of lead exposure on the expression of Uc.173 in nerve cells, we treated mouse neuroblastoma N2a cells with 0.1 um/L lead acetate and observed the changes of Uc.173 expression at various treatment time points. We tested the expression of Uc.173 in N2a cells at five time points (0 h, 12 h, 24 h, 36 h and 48 h) after the treatment and found with the extension of lead-exposure time, the expression of Uc.173 in N2a cells decreased gradually. Compared with 0 h, the expression of Uc.173 decreased by 2.4-fold in 48 h (Figure 3).

### Effect of Uc.173 on cell apoptosis

**Figure 4:**
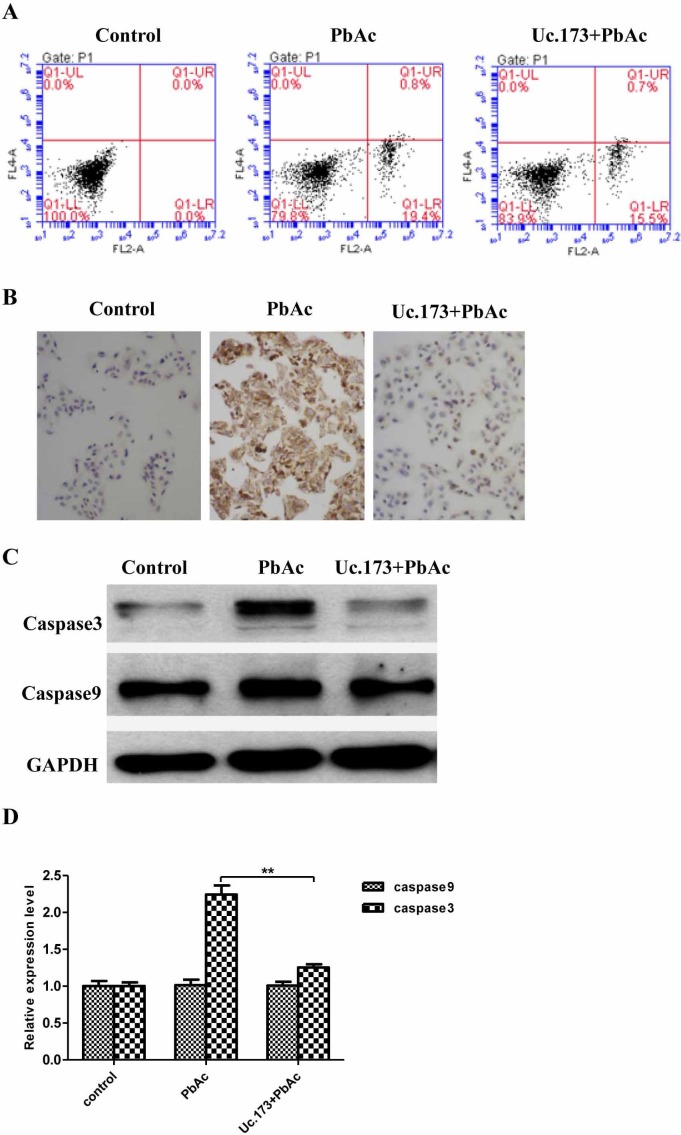
Detection of apoptotic markers **A**. shows apoptosis of the cells receiving different treatments detected by flow cytometry (FCM). Control represents transfection reagent control group, PbAc represents 48h lead exposure group and Uc.173+PbAc represents 6 h Uc.173 overexpression plus 48h lead exposure group. In the FCM diagrams, UL is necrotic cells, UR is necrotic late apoptotic cells, LL is live cells and LR is early apoptotic cells. **B**. shows the DNA breakage detected by TUNEL assay. As shown in the images which were 100 times amplified under a light microscope after hematoxylin counter stain, nuclei with fragmented DNA are represented by brown staining. Blue staining represents normal nuclei. **C**. shows the expression of caspase 3 and caspase 9 detected by western blot. GAPDH is the internal control. **D**. shows the relative expression levels corresponding to C. One-way ANOVA were used to compare control, lead exposure alone and Uc.173 overexpression plus lead exposure. The difference was highly statistically significant (**P < 0.01).

Studies have shown that the neurotoxicity of lead is associated with apoptosis of nerve cells. Whether Uc.173 effects lead-induced neuronal apoptosis has not been reported. We overexpressed Uc.173 in nerve cells using *in vitro* transcription and transfection and tested the apoptosis firstly at the cellular level. As shown in Figure 4A, the Q1-LR (Quadrant1-Low Right, representing early-stage apoptosis in a flow cytometry test) in the lead-treated alone group was 19.4%, and in the group of Uc.173 overexpression plus lead treatment, the Q1-LR was 15.5% (Figure 4A). Compared with being treated with lead alone, combining Uc.173 overexpression significantly reduced the apoptosis. This indicated that Uc.173 might have an apoptotic inhibitory function in lead-induced nerve injury.

We used the terminal deoxynucleotidyl transferase dUTP nick-end labeling (TUNEL) assay to further verify the anti-apoptotic function of Uc.173. TUNEL assay *in situ* detected fragmented DNA after lead treatment to evaluate cell apoptosis. As shown in Figure 4B, a large amount of nuclear DNAs were fragmented after 48-hour lead treatment, which indicated cells experienced significant apoptosis. Overexpressing Uc.173 for six hours combined with 48-hour lead treatment decreased DNA fragmentation significantly. The result of TUNEL assay also supported the anti-apoptotic effect of Uc.173 on lead-induced nerve injury.

We then tested the expression of caspase 3 and caspase 9, key members in the apoptotic pathway, by assessing protein levels with a western blot. We tested the control group, the lead-treatment alone group and the Uc.173-overexpressed plus lead-treatment group, respectively. As shown in Figure 4C and 4D, compared with the control group, the expression of caspase 3 in the lead-treatment alone group increased significantly. The overexpression of Uc.173 made the expression recover to a level similar to the control. However, the expression of caspase 9 in these three groups was not significantly different. Despite the fact that caspase 9 can regulate the expression of caspase 3, other mechanisms may also participate in the regulation of caspase 3. The difference in the expression of caspase 9 and caspase 3 in lead-induced nerve injury suggested other factors might affect the expression of caspase 3 during this process. In conclusion, we confirmed the anti-apoptotic function of Uc.173 in lead-induced nerve injury at the protein level.

### Identification of target miRNAs and genes

**Figure 5:**
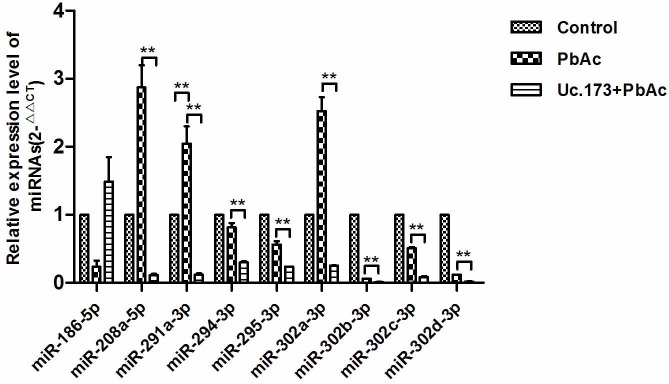
Expression of nine target miRNAs with highest potential in different groups Control represents the control group. PbAc represents 48 h lead exposure group. Uc.173+PbAc represent Uc.173 overexpression plus 48 h lead exposure group. U6 was used as an internal control in relative quantification. ** means *P* < 0.01, the difference was highly statistically significant.

Studies have shown that there is inter-regulation between T-UCR and miRNA. T-UCR can play a regulatory function through the mediation of miRNA [13]. To identify the potential target miRNAs of Uc.173, we used miRanda and TargetScan. Thirty-four miRNAs were predicted by both software (Table 1). To further screen the miRNAs, we analyzed the miRNAs using the RegRNA platform [14]. By analyzing the seed sequence, we obtained nine miRNAs which were most likely to be the target miRNAs of Uc.173 (miR-186-5p, miR-208a-5p, miR-291a-3p, miR-294-3p, miR-295-3p, miR-302a-3p, miR-302b-3p, miR-302c-3p and miR-302d-3p). We then used qPCR to validate their expression further. When compared with the lead-treatment alone group, qPCR suggested that eight miRNAs (miR-208a-5p, miR-291a-3p, miR-294-3p, miR-295-3p, miR-302a-3p, miR-302b-3p, miR-302c-3p and miR-302d-3p) in the Uc.173-overexpressed plus lead-treatment group were downregulated (Figure 5). Analysis of miRSystem revealed among the eight miRNAs, four miRNAs (miR-291a-3p, miR-295-3p, miR-302b-3p and miR-302d-3p) have apoptosis-associated target genes (*PI3K*, *NIK* and *Cn*) or a DNA-damage repair-associated target gene (*RAD23B*). These target genes are all key regulatory factors in the apoptotic pathway. Among these four miRNAs, only miR-291a-3p was upregulated in the lead-treatment alone group, in which the expression of Uc.173 was actually downregulated. Based on the bioinformatics analysis and the prediction of target genes, we propose that Uc.173 likely inhibits apoptosis through the mediation of miR-291a-3p, which needs further studies to verify.

**Table 1:** The target miRNAs of Uc.173 predicted by miRanda and TargetScan

miRNA ID	Accession	Site no.	Site type	Context+	Structure	Energy
mmu-miR-7078-3p	MIMAT0028063	1	7mer-m8,	0	140	−2.83
mmu-miR-7026-3p	MIMAT0027957	1	7mer-m8,	−0.258	141	−9.07
mmu-miR-5624-3p	MIMAT0022378	1	7mer-m8,	−0.304	145	−10.9
mmu-miR-7666-3p	MIMAT0029839	1	8mer-1a,	−0.503	141	−13.13
mmu-miR-466f-3p	MIMAT0004882	2	7mer-1a,7mer-m8,	−0.248	140	−5.95
mmu-miR-100-3p	MIMAT0017051	1	7mer-m8,	−0.238	152	−16.77
mmu-miR-511-3p	MIMAT0017281	1	7mer-m8,	−0.206	140	−9.48
*mmu-miR-302b-3p	MIMAT0003374	1	8mer-1a,	−0.439	151	−15.77
*mmu-miR-186-5p	MIMAT0000215	1	8mer-1a,	−0.168	150	−13.26
mmu-miR-7049-5p	MIMAT0028002	1	7mer-m8,	0	140	−12.91
mmu-miR-6540-5p	MIMAT0025585	1	7mer-m8,	−0.295	151	−12.18
mmu-miR-491-3p	MIMAT0017255	1	8mer-1a,	−0.309	150	−6.27
mmu-miR-215-3p	MIMAT0017169	1	8mer-1a,	−0.323	142	−21.22
mmu-miR-6932-5p	MIMAT0027764	1	8mer-1a,	−0.459	147	−16.99
mmu-miR-467b-5p	MIMAT0005448	1	8mer-1a,	−0.436	142	−9.72
*mmu-miR-302a-3p	MIMAT0000380	1	8mer-1a,	−0.428	147	−15.29
*mmu-miR-302c-3p	MIMAT0003376	1	7mer-m8,	−0.308	148	−15.74
*mmu-miR-208a-5p	MIMAT0017014	1	7mer-m8,	−0.133	148	−15.31
mmu-miR-8118	MIMAT0031424	1	8mer-1a,	−0.243	157	−11.59
mmu-miR-467f	MIMAT0005846	1	8mer-1a,	−0.27	140	−7.06
mmu-miR-195a-3p	MIMAT0017000	1	8mer-1a,	−0.185	142	−7.39
mmu-miR-6896-5p	MIMAT0027692	1	8mer-1a,	−0.402	140	−13.56
*mmu-miR-295-3p	MIMAT0000373	1	8mer-1a,	−0.428	146	−10.82
*mmu-miR-302d-3p	MIMAT0003377	1	8mer-1a,	−0.428	153	−18.23
mmu-miR-208b-5p	MIMAT0017280	1	7mer-m8,	−0.114	142	−7.35
mmu-let-7f-1-3p	MIMAT0004623	1	7mer-1a,	−0.046	143	−8.92
mmu-let-7a-1-3p	MIMAT0004620	1	7mer-1a,	−0.039	140	−7.78
mmu-miR-6415	MIMAT0025169	1	7mer-m8,	−0.114	154	−15.31
mmu-let-7c-2-3p	MIMAT0005439	1	7mer-1a,	−0.039	140	−7.78
mmu-miR-8094	MIMAT0031395	1	7mer-m8,	0	155	−18.71
mmu-miR-3572-3p	MIMAT0020636	1	8mer-1a,	−0.32	140	−10.28
*mmu-miR-294-3p	MIMAT0000372	1	8mer-1a,	−0.428	156	−12.54
*mmu-miR-291a-3p	MIMAT0000368	1	8mer-1a,	−0.428	152	−13.14
mmu-miR-152-5p	MIMAT0016991	1	8mer-1a,	−0.421	145	−16.85

* Represents nine target miRNAs with the highest potential predicted by miRanda and TargetScan, and analyzed using RegRNA platform.

## DISCUSSION

Lead is an important heavy metal in the environment. Exposure to lead threatens human health and can influence the intellectual development of children significantly, which mainly presents as learning and memory disorders [2]. Diagnosis of lead poisoning depends on biochemical indices, including blood lead, hair lead and urine lead. However, due to the limits of test tools, lead poisoning cannot be diagnosed until blood lead accumulates to a certain level and irreversible damage has already been generated [15]. The mechanisms of lead-induced nerve injury have been a hotspot for many years in the field of exogenous chemical-induced neurotoxicity, but the associated mechanisms are still not fully understood. Lots of studies indicate that lead-induced apoptosis is an important event during nerve injury caused by lead [16]. Current studies concentrate on the effects of protein-coding genes in this process; the roles of non-coding genes have been largely overlooked.

The majority of lncRNAs are transcribed by RNA polymerase II. The RNA molecules do not encode proteins due to the lack of open reading frames. LncRNAs are a kind of evolutionarily-conserved RNA molecule whose expression is dynamic and varies with the environment. Compared with protein-coding genes, lncRNAs have tissue and developmental specificity [17]. LncRNAs, including T-UCRs, have very important biological functions. The expressional type and amount of T-UCRs proved closely related to the survival rate of cancer patients [18]. T-UCRs can be used as markers for cancer diagnosis and prognosis, and have become potential targets for cancer therapy [19]. The functional mechanisms of lncRNAs are complicated and the role of lncRNAs in lead-induced nerve injury has not been reported. To explore whether T-UCRs may be involved in neuronal apoptosis induced by lead, we chose Uc.173, a T-UCR selected from a lncRNA chip in our previous screen, to study their molecular function in lead-induced neuronal apoptosis. We detected Uc.173 in a lead-induced mouse nerve injury model as well as in lead-exposed populations. The results showed that the expression of Uc.173 in the process of lead-induced nerve injury was significantly reduced.

Studies indicate that lead can induce the apoptosis of nerve cells in the hippocampus and cerebral cortex of developing and mature rats [20]. Cell apoptosis is a kind of programmed death controlled by genes [21], which is important for the maintenance of body homeostasis and the normal development of the nervous system. Apoptosis is also associated with diseases. Excessive apoptosis may harm normal nerve function. We explored the function of Uc.173 in lead-induced neural apoptosis from the cell, DNA and protein level. We treated N2a cells with lead acetate (PbAc) and found the expression of Uc.173 in the lead-exposed cells declined with time. We further overexpressed Uc.173 in N2a cells and then used FCM to detect the apoptosis at the cellular level and analyzed broken DNA using a TUNEL assay at the DNA level. The results of both methods suggested that apoptosis in lead-treated alone cells increased significantly. Overexpression of Uc.173 significantly weakened the apoptosis induced by lead treatment, which again favored the anti-apoptotic effect of Uc.173.

Cell apoptosis is regulated by a set of genes and is accompanied by changes in the expression of associated proteins [22], so we further investigated the anti-apoptotic function of Uc.173 at the protein level. The cascade reaction induced by the caspase cysteine protease family is the key event in the process of cell apoptosis. The activation of caspase is mediated by the mitochondrial dependent pathway and death receptor-mediated signaling pathway. The activated downstream caspases lead to cell apoptosis by cleaving specific substrates [23]. Caspase 3, a member of the caspase family which has been studied relatively thoroughly, is considered a key protease in apoptosis [24]. After being activated, it triggers a downstream cascade reaction to induce apoptosis. Caspase 3 exists in an inactive proenzyme before activation and is activated through being cleaved by multiple proteolytic enzymes [25]. Caspases can be activated through multiple ways; for example, caspase 9 can activate caspase 3, and caspase 3 in turn activates caspase 9 to form a positive feedback [25]. We used western blotting to detect the expression of caspase 3 and caspase 9 at the protein level. The expression of caspase 3 but not caspase 9 showed significant difference. This suggested Uc.173 may inhibit apoptosis by regulating the expression of caspase 3, and there should be other ways (not caspase 9) to activate caspase 3 in the process. In summary, we verified the anti-apoptotic function of Uc.173 from the cell, DNA and protein level.

Calin et al. [13] found that T-UCRs were associated with the negative regulation of miRNAs in leukemia and high-risk neuroblastoma cells and investigated the interaction of T-UCRs and miRNAs. A T-UCR is one of the lncRNAs, and researches show that lncRNAs play a role in regulating downstream target miRNA as ceRNA. They found T-UCRs and miRNAs could inter-regulate each other. Mestdagh et al. [26] deeply studied the initiation and regulation of T-UCR and miRNA transcription and found both of the non-coding RNAs had similar transcription characteristics. Jiang et al. [18] found the expression of some T-UCRs was negatively correlated with miRNA expression. Scaruffi et al. [27] suggested that a deregulation of the miRNA/T-UCR network may play an important role in the pathogenesis of neuroblastoma. Sana J et al. [11] found uc.73 to influence apoptosis in colon cancer cells, and uc.338 to inhibit the growth of hepatocellular carcinoma cells. Using target gene prediction plus qPCR testing, we discovered that in apoptosis induced by lead, Uc.173 potentially regulated apoptosis through the mediation of miR-291a-3p.

In conclusion, Uc.173, a T-UCR, plays an important role in lead-induced neuronal apoptosis. Our study reveals a new mechanism of lead-induced nerve injury.

## MATERIALS AND METHODS

### Construction of lead-induced mouse brain injury model

A batch of 60 ablactating C57bl/6 mice (three weeks old) with a weight range of 10-12 g were purchased from the Medical Animal Experimental Center of Guangdong, China and fed in the specific pathogen free (SPF) animal room of Laboratory Animal Center of Guangzhou Medical University, China. The temperature was 20-26°C and the relative humidity was 40%-70%. The light/dark cycle was 12/12 hours. After a three-day adaptation, the C57bl/6 mice were randomly divided into control group (sterile water, *n* = 30) and lead acetate-treatment group (lead acetate solution, *n* = 30). Each cage fed five mice. The mice in the lead acetate-treatment group were fed with drinking water containing 9.6 mmol/L lead acetate. The mice in the two groups were sacrificed after 1, 2 and 5 weeks treatment of lead acetate (ten mice/group/time point). All processes involving experimental animals were approved by the Animal Care and Use Committee of Guangzhou Medical University. The whole blood of the mice was collected and blood lead concentration was tested by Agilent 7700C ICP-MS (Agilent, Santa Clara, CA, USA). Pathological examination by HE staining was performed on sacrificed mice in the two groups.

### Collection of the serum samples of lead-exposed population

Study subjects were the students from an elementary school in Guangzhou City. The area where the school is located may be lead polluted due to the intensive distribution of lead-associated plants. A total of 200 students in grade 1, 2 or 3 took the blood lead test. In the meantime, the serum samples were collected for Uc.173 detection. All the students' parents signed the informed consent document. We isolated serum and detected blood lead concentration in December, 2014. We asked the children subsumed about their medical history and excluded children with poor compliance. Children with low blood lead concentration (less than 20 ng/L) and with high blood lead concentration (more than 80 ng/L) were paired up for the study.

### Construction of lead-exposed nerve cell model

The cellular model was constructed by treating mouse N2a nerve cells with 0.1 μm/L lead acetate solution for 48 hours.

### Extraction of RNA

The total RNAs (including lncRNAs) of mouse hippocampus tissue were extracted using TRIzol Reagent (Invitrogen, Life Technologies, Carlsbad, CA, USA). The hippocampus tissue was placed in a 1.5 ml centrifuge tube containing 300 μl TRIzol reagent and ground to a paste using an electric micro-tissue homogenizer. TRIzol (700 μl) was added and the grinding continued. After this, the homogenate samples were placed at room temperature for 5 minutes to completely separate the DNA-protein complex. The extraction of total RNAs was performed by following the manufacturer's protocol. The quality and concentration of extracted RNAs were measured by a NanoDrop1000 Spectrophotometer (NanoDrop Technologies, Wilmington, DE, USA). The total RNAs in mouse serum and human serum were extracted using TRIzol LS (Invitrogen). TRIzol LS Reagent (300 μl; Invitrogen) was added into 100 μl serum and fully mixed. After placing at room temperature for 5 minutes, the total RNAs were extracted according to the TRIzol LS manual.

### Quantitative real-time PCR (qRT-PCR)

A Prime-Script RT reagent Kit (TaKaRa, Dalian, China) was used to reverse transcribe RNAs to cDNAs. PCR was performed using SYBR Premix Ex Taq (TaKaRa). The primers of internal control genes and specific primers are shown in Supplementary Table 1. The calculation method for relative expression amount in the animal model and human was 2^−ΔCt^, and in the cellular model it was 2^−ΔΔCt^.

### Construction and transfection of overexpression vector

Extraction of genomic DNA, culture of receptor bacteria and preparation of competent cells were performed before the construction of the vector. The construction of the vector included the amplification of target fragments, linkage of amplified fragments with pGEM-T Easy-Uc.173 (Biosense, Guangzhou, China) vectors and transfection of competent cells. Transformation: 200 μL of competent cell suspension, previously stored at −80°C, was thawed in an ice bath. Ligation products were added, mixed gently and placed on ice for 30 minutes. The cells were heat shocked for 80 seconds in a water bath at 42°C and immediately placed on ice for 3-5 minutes. One milliliter LB liquid medium (Not containing ampicillin (Amp)), was added to the tube, mixed and incubated for 1 hour at 37°C. One hundred micro liters of bacterial solution was spread onto the screen plate containing Amp and incubated at 37°C for 16-24 hours. Positive clones were analyzed using electrophoresis and then sequenced and transcribed to RNA. The transfection system contained: 12 μl EndoFectin-Lenti (Gene Copoeia, Rockville, MD, USA) 1 μg Uc.173, 200 μl Dulbecco minimum essential medium (DMEM) without serum. Twenty-four hours after transfection, PCR was performed to evaluate transfection efficiency.

### Detection of apoptosis by flow cytometry (FCM)

Annexin V-Fluorescein Isothiocyanate Apoptosis Detection Kit (KeyGen Biotech, Nanjing, China) was used to detect cell apoptosis. The cells were digested by non-ethylene diamine tetra acetic acid (EDTA)-containing trypsin 48 hours after transfection and the treatment of lead, and then centrifuged (300 × *g*, 4°C, 5 min). Approximately 1-5 × 10^5^ cells were collected and resuspended in 100 μl 1×Binding Buffer (KeyGen Biotech). Annex in V-FITC (5 μl KeyGen Biotech) and 5 μl PI Staining Solution (KeyGen Biotech) were added and mixed gently. The reaction lasted for 10 minutes under room-temperature in a light-proof environment. Four hundred micro liters of 1×Binding Buffer was added and mixed gently. FCM was performed within one hour using a flow cytometer (BD Biosciences, NJ, USA). Annexin V-FITC+PI- quadrant represented early apoptotic cells; Annexin V-FITC+PI- quadrant represented early apoptotic cells (LR); Annexin V-FITC-PI+ quadrant represented necrotic cells (UL); Annexin V-FITC+PI+ quadrant represented late apoptotic and necrotic cells (UR); Annexin V-FITC-PI- quadrant represented live cells (LL). Finally, the ratio of apoptotic cells accounting for total cells was calculated.

### Detection of apoptosis by TUNEL assay

TUNEL apoptosis detection kit (Roche, Basel, Switzerland) was used to detect apoptosis. The cells were adherently cultured on a Lab-Tek slide. After treatment with lead acetate, the slide was washed twice with PBS. The sample was fixed using 4% polyformaldehyde at room temperature for 20 minutes and washed twice (5 minutes each time) with PBS. The prepared TUNEL reaction mixture was dropped onto the cover slip and covered by a cover glass. The section was incubated at 37°C for 60 minutes and washed in PBS for three times (5 minutes each time). Converter-Peroxidase (POD) solution was added and incubated at 37°C for 30 minutes, and then washed in PBS for three times (5 minutes each time). Staining was performed using Diaminobenzidine (DAB) stain kit (Roche) at room temperature for 10 minutes, and was then washed in PBS for 5 minutes three times. The section was counterstained with hematoxylin for ten seconds and rinsed with water. The section was dehydrated with gradient alcohol, then dried and mounted with neutral gum. The section was observed under an optical microscope. Brown-stained nuclei represented positive staining. Blue-stained nuclei represented counterstaining by hematoxylin.

### Detection of apoptotic proteins by western blot

A total protein extraction kit (KeyGen Biotech, Nanjing, China) was used to extract total protein. Procedures were followed according to the kit manual. The OD (optical density) values of the samples were measured using Coomassie Brilliant Blue G250. The concentrations of the protein of the samples were adjusted to be the same level (4-8 μg/μl). Bio-Rad (Hercules, California, USA) standard wet transfer apparatus and Poly vinylidene fluoride membrane (PVDF) (Merck Millipore, Darmstadt, German) were used to transfer the proteins from the gel to the membrane. A constant flow (200 mA) was applied in the process of transfer and the transfer time was 60-120 minutes according to the molecular weight of the target proteins. The membrane was placed into TBST after transfer and rinsed for 1-2 minutes. Blocking buffer, which contained 5% skimmed milk powder, was added to block at room temperature for 60 minutes. The PVDF membrane was incubated in blocking buffer which contained corresponding primary antibody at 4°C overnight. The membrane was washed with TBST three times for 5 minutes each. Then the PVDF membrane was incubated in blocking buffer which contained the diluted secondary antibody at room temperature for 1 hour, then washed with TBST three times for 15 minutes each. A Beyo ECL Plus chemiluminescence kit (Beyotime, Shanghai, China) was used for staining in a darkroom. Finally, routine exposure and development were performed using X-ray film.

### MiRNA prediction and detection

MiRanda and TargetScan were used to predict the target miRNAs of Uc.173. Default parameters of the software were applied and the results were not filtered. Only miRNAs predicted by both software were reserved and analyzed using the RegRNA platform. The expression of the miRNAs was detected by qRT-PCR. The 2-^ΔΔCT^ method was used for relative quantification.

### Data analysis

SPSS17.0 (SPSS Inc., Chicago, IL, USA) was used for data analysis. One-way ANOVA was used to compare the following three differences: the expression of Uc.173 in lead-exposed N2a cells, the gray values of the expression of the proteins, and the expression of miRNAs in lead-exposed nerve cells. A paired t-test was used to compare the difference of lead concentration in tissue or serum in the lead-induced nerve injury animal model. A Mann-Whitney test was used to compare the difference of serum lead concentration in different populations. A value of *P* < 0.05 represents a statistically significant difference and *P* < 0.01 represents a highly statistically significant difference.

## SUPPLEMENTARY MATERIAL TABLE



## References

[1] Muller D, Nemitz R, Koch RD (1984). Diagnosis of lead-induced occupational nerve damage [Article in German]. Z Gesamte Hyg.

[2] Chang J, Kueon C, Kim J (2014). Influence of lead on repetitive behavior and dopamine metabolism in a mouse model of iron overload. Toxicol Res.

[3] Kirel B, Aksit MA, Bulut H (2005). Blood lead levels of maternal-cord pairs, children and adults who live in a central urban area in Turkey. Turk J Pediatr.

[4] Hu G, Yang T, Zheng J, Dai J, Nan A, Lai Y, Zhang Y, Yang C, Jiang Y (2015). Functional role and mechanism of lncRNA LOC728228 in malignant 16HBE cells transformed by anti-benzopyrene-trans-7,8-dihydrodiol-9,10-epoxide. Mol Carcinog.

[5] Chen YA, Aravin AA (2015). Non-Coding RNAs in Transcriptional Regulation. Curr Mol Biol Rep.

[6] Pilyugin M, Irminger-Finger I (2014). Long non-coding RNA and microRNAs might act in regulating the expression of BARD1 mRNAs. Int J Biochem Cell Biol.

[7] Liang WC, Fu WM, Wong CW, Wang Y, Wang WM, Hu GX, Zhang L, Xiao LJ, Wan DC, Zhang JF, Waye MM (2015). The lncRNA H19 promotes epithelial to mesenchymal transition by functioning as MiRNA sponges in colorectal cancer. Oncotarget.

[8] Lomonaco V, Martoglia R, Mandreoli F, Anderlucci L, Emmett W, Bicciato S, Taccioli C (2014). UCbase 2.0: ultraconserved sequences database (2014 update). Database (Oxford).

[9] Fassan M, Dall'Olmo L, Galasso M, Braconi C, Pizzi M, Realdon S, Volinia S, Valeri N, Gasparini P, Baffa R, Souza RF, Vicentini C, D'Angelo E (2014). Transcribed ultraconserved noncoding RNAs (T-UCR) are involved in Barrett's esophagus carcinogenesis. Oncotarget.

[10] Scaruffi P, Stigliani S, Moretti S, Coco S, De Vecchi C, Valdora F, Garaventa A, Bonassi S, Tonini GP (2009). Transcribed-ultra conserved region expression is associated with outcome in high-risk neuroblastoma. BMC Cancer.

[11] Sana J, Hankeova S, Svoboda M, Kiss I, Vyzula R, Slaby O (2012). Expression levels of transcribed ultraconserved regions uc.73 and uc.388 are altered in colorectal cancer. Oncology.

[12] Watters KM, Bryan K, Foley NH, Meehan M, Stallings RL (2013). Expressional alterations in functional ultra-conserved non-coding RNAs in response to all-trans retinoic acid—induced differentiation in neuroblastoma cells. BMC Cancer.

[13] Calin GA, Liu CG, Ferracin M, Hyslop T, Spizzo R, Sevignani C, Fabbri M, Cimmino A, Lee EJ, Wojcik SE, Shimizu M, Tili E, Rossi S (2007). Ultraconserved regions encoding ncRNAs are altered in human leukemias and carcinomas. Cancer Cell.

[14] Huang HY, Chien CH, Jen KH, Huang HD (2006). RegRNA: an integrated web server for identifying regulatory RNA motifs and elements. Nucleic Acids Res.

[15] Naujokas MF, Basta NT, Cheng Z, Hettiarachchi GM, Maddaloni M, Schadt C, Scheckel KG, Attanayake C, Henry H (2015). Bioavailability-based *in situ* remediation to meet future lead (Pb) standards in urban soils and gardens. Environ Sci Technol.

[16] Olson L, Bjorklund H, Henschen A, Palmer M, Hoffer B (1984). Some toxic effects of lead, other metals and antibacterial agents on the nervous system—animal experiment models. Acta Neurol Scand Suppl.

[17] Francescatto M, Vitezic M, Heutink P, Saxena A (2014). Brain-specific noncoding RNAs are likely to originate in repeats and may play a role in up-regulating genes in cis. Int J Biochem Cell Biol.

[18] Peng JC, Shen J, Ran ZH (2013). Transcribed ultraconserved region in human cancers. RNA Biol.

[19] Scaruffi P (2011). The transcribed-ultraconserved regions: a novel class of long noncoding RNAs involved in cancer susceptibility. SCI WORLD J.

[20] Chao SL, Moss JM, Harry GJ (2007). Lead-induced alterations of apoptosis and neurotrophic factor mRNA in the developing rat cortex, hippocampus, and cerebellum. J Biochem Mol Toxicol.

[21] Hoang TM, Moghaddam L, Williams B, Khanna H, Dale J, Mundree SG (2015). Development of salinity tolerance in rice by constitutive-overexpression of genes involved in the regulation of programmed cell death. Front Plant Sci.

[22] Capoccia E, Cirillo C, Marchetto A, Tiberi S, Sawikr Y, Pesce M, D'Alessandro A, Scuderi C, Sarnelli G, Cuomo R, Steardo L, Esposito G (2015). S100B-p53 disengagement by pentamidine promotes apoptosis and inhibits cellular migration via aquaporin-4 and metalloproteinase-2 inhibition in C6 glioma cells. ONCOL LETT.

[23] Hamacher-Brady A, Brady NR (2015). Bax/Bak-dependent, Drp1-independent targeting of XIAP into inner- mitochondrial compartments counteracts smac-dependent effector caspase activation. J Biol Chem.

[24] Fang X, Miao XL, Liu JL, Zhang DW, Wang M, Zhao DD, Mu QQ, Yu N, Mo FF, Yin HP, Gao SH (2015). Berberine induces cell apoptosis through cytochrome C/apoptotic protease-activating factor 1/caspase-3 and apoptosis inducing factor pathway in mouse insulinoma cells. Chin J Integr Med.

[25] Yiang GT, Yu YL, Lin KT, Chen JN, Chang WJ, Wei CW (2015). Acetaminophen induces JNK/p38 signaling and activates the caspase-9-3-dependent cell death pathway in human mesenchymal stem cells. Int J Mol Med.

[26] Mestdagh P, Fredlund E, Pattyn F, Rihani A, Van Maerken T, Vermeulen J, Kumps C, Menten B, De Preter K, Schramm A, Schulte J, Noguera R, Schleiermacher G (2010). An integrative genomics screen uncovers ncRNA T-UCR functions in neuroblastoma tumours. Oncogene.

[27] Scaruffi P, Stigliani S, Coco S, Valdora F, De Vecchi C, Bonassi S, Tonini GP (2010). Transcribed-ultra conserved region expression profiling from low-input total RNA. BMC Genomics.

