# Supplementary material for: A transcribed ultraconserved noncoding RNA, Uc.173, is a key molecule for the inhibition of lead-induced neuronal apoptosis
Source: Oncotarget. 2015 Dec 13;7(1):112–24. doi: 10.18632/oncotarget.6590 (PMC4807986; doi:10.18632/oncotarget.6590)
Supplement: Supplementary file 1 [file oncotarget-07-0112-s001.pdf]

A transcribed ultraconserved noncoding RNA, Uc.173, is a key molecule for the inhibition of lead-induced neuronal apoptosis

Supplementary Material

Supplementary Table1. qPCR primers and their sequences

Gene	Primer Sequence
Uc.173-F	ACTTTTTATTGCATGGTGTGAACT
Uc.173-R	CACTTGGAAAAAAATACAAACAGG
GADPH-F	AGGCCGGTGCTGAGTATGTC
GAPDH-R	TGCCTGCTTCACCACCTTCT
mmu-miR-186-5p	CAAAGAATTCTCCTTTTGGGCT
mmu-miR-208a-5p	GAGCTTTTGGCCCGGGTTATAC
mmu-miR-291a-3p	AAAGTGCTTCCACTTTGTGTGC
mmu-miR-294-3p	AAAGTGCTTCCCTTTTGTGTGT
mmu-miR-295-3p	AAAGTGCTACTACTTTTGAGTCT
mmu-miR-302a-3p	TAAGTGCTTCCATGTTTTGGTGA
mmu-miR-302b-3p	TAAGTGCTTCCATGTTTTAGTAG
mmu-miR-302c-3p	AAGTGCTTCCATGTTTCAGTGG
mmu-miR-302d-3p	TAAGTGCTTCCATGTTTGAGTGT
U6	CTCGCTTCGGCAGCACA
